# SPARTA: Simple Program for Automated reference-based bacterial RNA-seq Transcriptome Analysis

**DOI:** 10.1186/s12859-016-0923-y

**Published:** 2016-02-04

**Authors:** Benjamin K. Johnson, Matthew B. Scholz, Tracy K. Teal, Robert B. Abramovitch

**Affiliations:** Department of Microbiology and Molecular Genetics, Michigan State University, East Lansing, MI 48824 USA; VANTAGE, Vanderbilt University, Nashville, TN 37235 USA; Data Carpentry, ᅟ, ᅟ

**Keywords:** Bioinformatics, Data analysis, Transcriptomics, Microbiology, Next-generation sequencing, High-throughput sequencing

## Abstract

**Background:**

Many tools exist in the analysis of bacterial RNA sequencing (RNA-seq) transcriptional profiling experiments to identify differentially expressed genes between experimental conditions. Generally, the workflow includes quality control of reads, mapping to a reference, counting transcript abundance, and statistical tests for differentially expressed genes. In spite of the numerous tools developed for each component of an RNA-seq analysis workflow, easy-to-use bacterially oriented workflow applications to combine multiple tools and automate the process are lacking. With many tools to choose from for each step, the task of identifying a specific tool, adapting the input/output options to the specific use-case, and integrating the tools into a coherent analysis pipeline is not a trivial endeavor, particularly for microbiologists with limited bioinformatics experience.

**Results:**

To make bacterial RNA-seq data analysis more accessible, we developed a Simple Program for Automated reference-based bacterial RNA-seq Transcriptome Analysis (SPARTA). SPARTA is a reference-based bacterial RNA-seq analysis workflow application for single-end Illumina reads. SPARTA is turnkey software that simplifies the process of analyzing RNA-seq data sets, making bacterial RNA-seq analysis a routine process that can be undertaken on a personal computer or in the classroom. The easy-to-install, complete workflow processes whole transcriptome shotgun sequencing data files by trimming reads and removing adapters, mapping reads to a reference, counting gene features, calculating differential gene expression, and, importantly, checking for potential batch effects within the data set. SPARTA outputs quality analysis reports, gene feature counts and differential gene expression tables and scatterplots.

**Conclusions:**

SPARTA provides an easy-to-use bacterial RNA-seq transcriptional profiling workflow to identify differentially expressed genes between experimental conditions. This software will enable microbiologists with limited bioinformatics experience to analyze their data and integrate next generation sequencing (NGS) technologies into the classroom. The SPARTA software and tutorial are available at sparta.readthedocs.org.

## Background

One of the most common applications of RNA sequencing (RNA-seq) is to identify differentially expressed genes under differing experimental conditions. Before biological insights can be gained, one must process and analyze the large datasets generated from each sequencing experiment. Each sample contains millions of reads that must be trimmed and assessed for read quality, mapped back to a reference genome (or assembled *de novo* in the absence of a reference), counted for transcript abundance, and tested for differential gene expression. Many computational analysis tools have been developed specifically to work with RNA-seq data; however, a single tool is often not suitable and requires several different applications assembled into a workflow. This task can be complicated as both the tool choice and input and output file formats for a given tool need to be considered and potentially modified to meet the requirements for the subsequent analysis step. Several RNA-seq analysis workflows exist, however, most are designed for eukaryotic organisms [1–11]. The goal of this work is to assemble several open-source computational tools to deliver a complete, accessible, and easy-to-use reference-based bacterial RNA-seq analysis workflow that is amenable to both the research laboratory and undergraduate classroom.

## Implementation

**Fig. 1:**
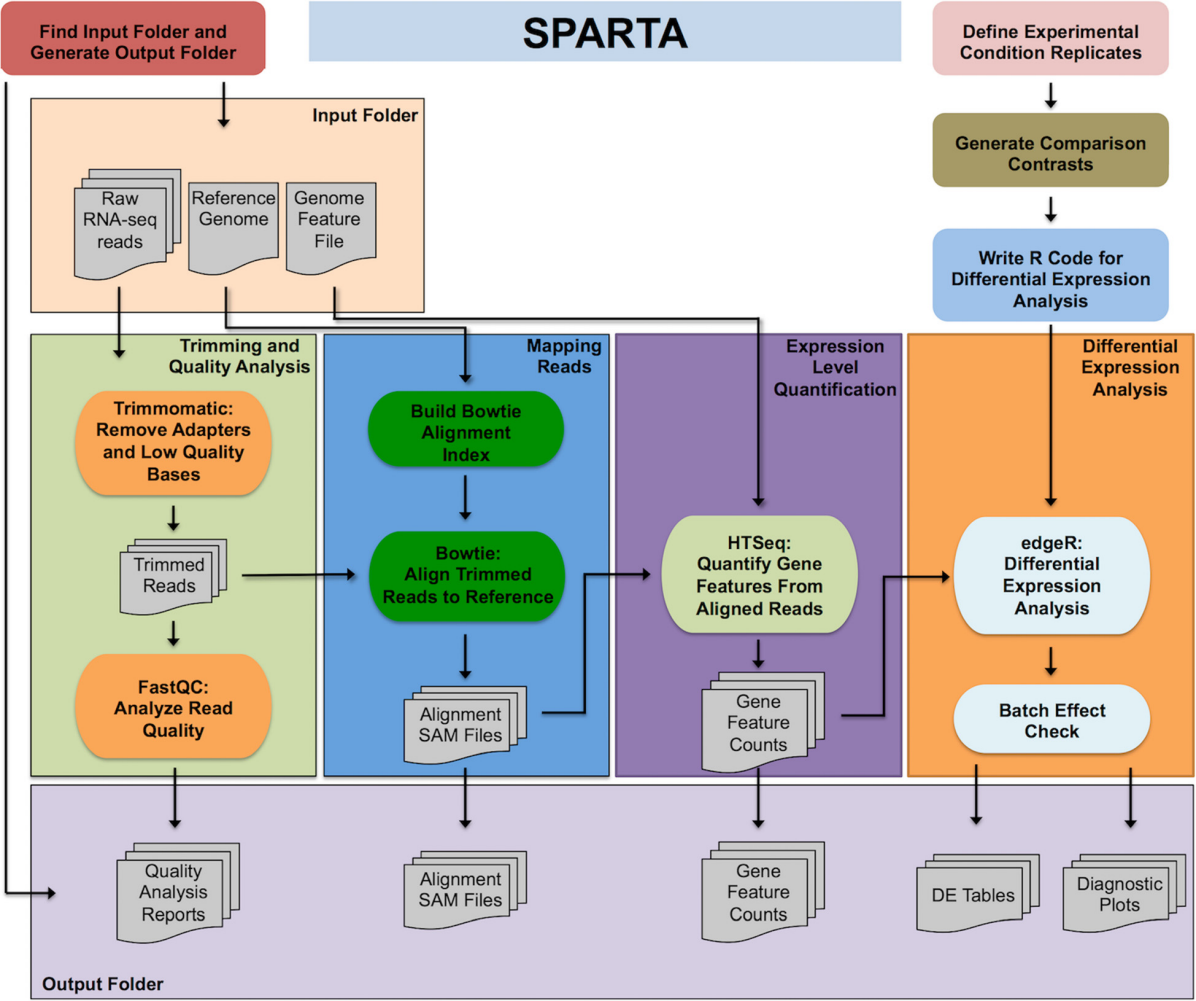
SPARTA workflow diagram. Single-end Illumina FASTQ files, a FASTA formatted reference genome, and genome feature file (gff or gtf) are given as inputs to the workflow. Trimmomatic and FastQC perform trimming of adapters and low quality bases/reads and quality assessment reports, respectively. Bowtie maps the trimmed reads to the reference. HTSeq quantifies transcript abundance. R/edgeR tests for statistically significant genes and warns the user of potential batch effects present in the analyzed data set

The SPARTA workflow (Fig. 1) is implemented utilizing Python for file input/output management and tool execution, combining several open-source computational tools. The SPARTA workflow analyzes data by: conducting read trimming and adapter removal with Trimmomatic [12]; performing quality analysis of the data sets with FastQC [13]; mapping the reads to the reference with Bowtie [14]; counting transcript or gene feature abundance with HTSeq [15]; and, analyzing differential gene expression with edgeR [16–18]. Within the differential gene expression analysis step, batch effects can be detected and the user is warned that potentially unintended variables need to be considered. If left unaccounted for, batch effects can significantly skew the results of the data analysis, leading to inappropriate experimental conclusions [19]. Following analysis, SPARTA outputs quality analysis reports, gene feature counts and differential gene expression tables and scatterplots.

SPARTA requires Python 2, NumPy (a Python library for numerical analyses), Java and R. Once Python is installed, the user initializes SPARTA, which then checks for the necessary dependencies at runtime. If any of these dependencies are not met, SPARTA informs the user of the missing components. To reduce complex software installation, SPARTA is distributed with the required software and an online tutorial (http://sparta.readthedocs.org) guides the user through installation and data analysis procedures for each operating system platform. The workflow maintains analytic flexibility for specific use cases by allowing the user to tailor the options utilized for each analysis step, but can proceed without requiring option specification. Further, SPARTA will write the necessary R commands at runtime and will generate the appropriate contrasts to test all possible comparisons between user defined experimental conditions. The workflow is distributed with an example data set containing the first 100,000 reads from a previously published study [20]. This data set is included to allow the user to become rapidly familiarized with the analysis procedure as well as ensure the appropriate dependencies are met.

## Results and discussion

RNA-seq transcriptional profiling is becoming increasingly routine, and there is a demand for applications such as SPARTA that enable stand-alone workflows. Though several bacterial RNA-seq analysis workflows have been developed [4, 5, 11], SPARTA is currently the only workflow capable of addressing the possibility of batch effects within the data set as well as the other necessary analysis procedures to identify differentially expressed genes. Using a previously published data set [20], SPARTA was capable of analyzing 4 experimental conditions containing 8 samples with approximately 30 million reads per sample in 4 h on an off-the-shelf iMac computer (8 GB RAM, Intel i5 2.7GHz quad-core processor). SPARTA can also be implemented in high performance computing environments utilizing the non-interactive mode functionality.

As NGS technologies and applications continue to permeate life science research, undergraduate education must incorporate the use of contemporary sequencing techniques to address biological questions. However, despite the rapid increase in data intensive experimental biology, undergraduates receiving a life sciences degree are often not exposed to the tools and basic computational skills required to study NGS data sets. To address this shortcoming, we have developed an online tutorial to guide students through the RNA-seq analysis process (http://sparta-teaching.readthedocs.org). The SPARTA teaching tool was integrated into a senior level genomics course and successfully engaged students in the theory and application of RNA-seq data analysis.

**Fig. 2:**
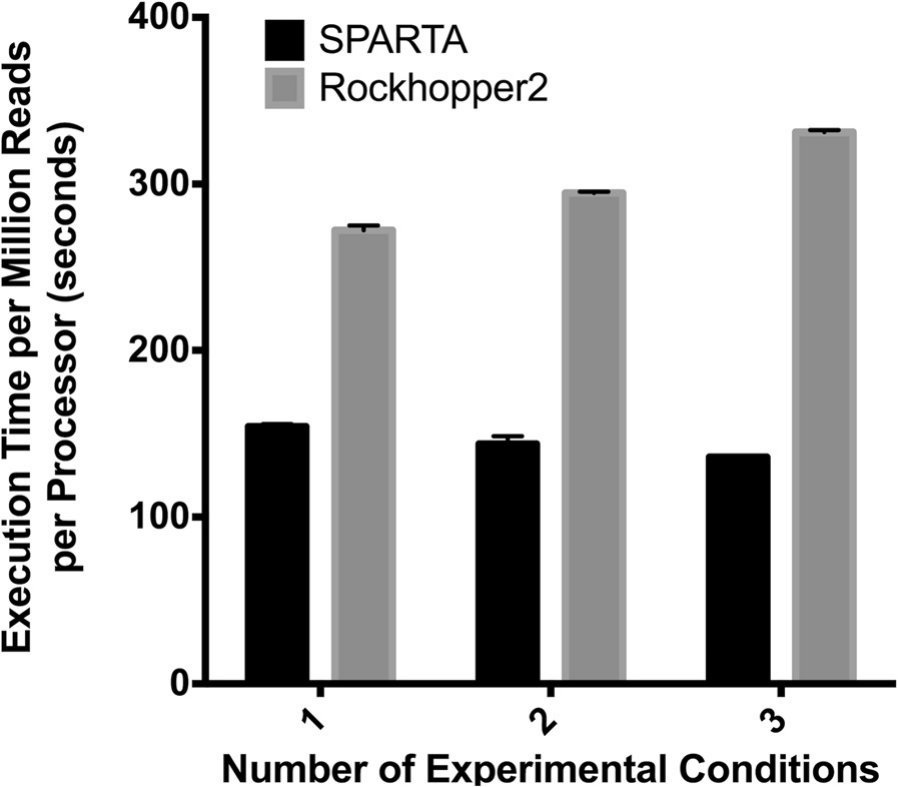
Data analysis execution time comparison between SPARTA and Rockhopper2. The two programs were compared for execution time when processing *one*, *two*, or *three* experimental conditions as compared to a reference condition. Both SPARTA (1.0) and Rockhopper2 (2.03) were installed and tested on an off-the-shelf iMac (2.7 GHz i5, 8 GB memory, OSX 10.11.2). Dependencies: Java (1.6.0_65), Python (2.7.9), and R (3.2.2). Data are the mean of three software executions and *error bars* represent the standard deviation. Data files (100,000 reads/file) utilized were the example data bundled with SPARTA

SPARTA and Rockhopper2 are both bacterial RNA-seq workflows that provide similar features [5]. An execution time comparison was conducted between the two platforms. SPARTA was executed with default parameters and Rockhopper2 was tested with default parameters, verbose output, SAM output, and operon and untranslated region identification turned off. Further, the Rockhopper2 cache was cleared before each test to mimic a first time analysis. When SPARTA was compared to Rockhopper2 for execution, SPARTA exhibited greater scalability (Fig. 2). Therefore, differentiating features of SPARTA as compared to Rockhopper2 include: improved scalability; incorporation of trimming and quality control of reads; and, a check for potential batch effects within the data set. Notably, Rockhopper2 provides additional functions not provided by SPARTA, such as operon analysis, definition of untranslated regions, and files for visualization of the results in a genome browser.

### Future directions and functionality

Additional features and functionality that will be incorporated into future releases of SPARTA are listed below and will be updated at http://sparta.readthedocs.org/en/latest/wishlist.html. Further, to become involved into the active development of SPARTA, the current state of the code base and feature development can be found on GitHub through http://sparta.readthedocs.org under the “Contribute” heading. Future releases of SPARTA will include but not limited to: 1) automated batch effect correction, 2) additional input file format support, 3) paired-end reads support, 4) read mapping output with normalized expression values, 5) operon analysis and definition of untranslated regions, 6) files for visualization in common genome browsers, and 7) reference-free transcriptome analysis.

## Conclusions

SPARTA is a bacterial RNA-seq analysis tool capable of taking raw Illumina reads to differentially expressed genes in a turn-key, stand-alone workflow format that takes advantage of existing state of the art analysis tools and warns the user of potential batch effects. By reducing the required computational proficiency to perform transcriptional profiling experiments using RNA-seq, SPARTA can enable microbiologists to accelerate their research and provide instructors the ability to incorporate a hands-on approach to NGS technologies in the classroom. Further, SPARTA maintains analytic flexibility by allowing the user to tailor the analysis through option specification but is capable of proceeding with default values.

## Availability and requirements

**Project name**: SPARTA.

**Project home page**: http://sparta.readthedocs.org; http://sparta-teaching.readthedocs.org.

**Operating system**: Platform independent.

**Programming language**: Python.

**Other requirements**: Java and R.

**License**: Creative Commons BY version 4 or greater.

## Abbreviations

NGS: next generation sequencing
RNA-seq: RNA sequencing
